# Medical Miscellany

**Published:** 1848

**Authors:** 


### ARTICLE III.

### MEDICAL MISCELLANY.

*The Chicago Marine Hospital*, for which $10,000 was appropriated by the last session of Congress, we understand, is to be erected upon the grounds belonging the Government near the south pier in the city, and that the construction of the buildings will be prosecuted vigorously until completed. This is a most important and praiseworthy object, upon which twice the amount of money appropriated might be judiciously expended.

*The Ohio Medical and Surgical Journal.*—The first number of this new cotemporary, under the editorial charge of Prof. Butterfield was issued on the first of September last, but was received too late for notice in our last number. It is issued every other month, and is of the same size and price of our own. The number for November is also received, and like the first, is full of interesting matter. We like the tone of independence manifested by the editor, and most heartily wish him success in his arduous undertaking.

*The St. Louis Medical and Surgical Journal.*—The two Medical Journals of St. Louis have been united, and the resulting journal with the above title is under the joint management of the editors of both original periodicals. We are glad to see this evidence of friendly feeling between the faculties in two rival medical schools situated in the same town. It speaks well for both, and we have no doubt will be a more profitable arrangement than the former one so far as the publishers are concerned.

